# Enxerto de Bypass de Artéria Coronária Guiado por Angiografia ou Fisiologia: Uma Metanálise

**DOI:** 10.36660/abc.20200763

**Published:** 2021-11-22

**Authors:** José Martins, Vera Afreixo, Luís Santos, Luís Fernandes, Ana Briosa

**Affiliations:** 1 Baixo Vouga Hospital Centre Aveiro Portugal Baixo Vouga Hospital Centre, Aveiro - Portugal; 2 University of Aveiro CIDMA/IBIMED/Department of Mathematics Aveiro Portugal CIDMA/IBIMED/Department of Mathematics, University of Aveiro, Aveiro - Portugal; 3 University of York Centre for Health Economics York Reino Unido Centre for Health Economics, University of York, York - Reino Unido

**Keywords:** Doença da Artéria Coronariana, Angiografia, Metanálise, Artéria Coronária/fisiologia, Angiografia Coronária, Ponte de Artéria Coronária

## Abstract

**Fundamento:**

Enquanto a angiografia coronária invasiva é considerada padrão outro para o diagnóstico da doença arterial coronariana (DAC), envolvendo os vasos coronários epicárdicos, a revascularização coronariana guiada por fisiologia representa uma prática padrão ouro contemporânea para a administração invasiva de pacientes com DAC intermediária. Porém, os resultados de longo prazo da avaliação da gravidade da estenose por meio da fisiologia, em comparação à angiografia como guia para a cirurgia de bypass – enxerto de bypass de artéria coronária (CABG), ainda são incertos. Esta metanálise visa avaliar os resultados clínicos de um CABG guiado por fisiologia em comparação a um CABG guiado pela angiografia.

**Objetivos:**

Buscamos determinar se os resultados entre um CABG guiado por fisiologia e os de um CABG guiado por angiografia são diferentes entre si.

**Métodos:**

Pesquisamos nas bases Medline, EMBASE e Cochrane Library. A última data de busca foi junho de 2020, e todos os estudos anteriores foram incluídos. Realizamos uma metanálise de razão de risco agrupado para quatro principais resultados: morte por todas as causas, infarto do miocárdio (IM), revascularização do vaso alvo (TVR) e eventos cardiovasculares adversos maiores (MACE). Valor de p <0,05 foi considerado estatisticamente significante. A heterogeneidade foi avaliada com o teste Q de Cochran, e quantificada pelo índice I2.

**Resultados:**

Identificamos cinco estudos incluindo um total de 1.114 pacientes. Uma metanálise agrupada não demonstrou diferenças significativas entre a estratégia da fisiologia e da angiografia para IM (razão de risco [RR] = 0,72; IC95%, 0,39–1,33; I2 = 0%; p = 0,65), TVR (RR = 1,25; IC95% = 0,73–2,13; I2 = 0%; p = 0,52), ou MACE (RR = 0,81; IC95% = 0,62–1,07; I2 = 0%; p = 1). A estratégia da fisiologia apresentou 0,63 vezes o risco de morte por todas as causas em comparação à estratégia da angiografia (RR = 0,63; IC95% = 0,42–0,96; I2 = 0%; p = 0,55).

**Conclusão:**

Esta metanálise demonstrou uma redução nas mortes por todas as causas quando usada a estratégia do CABG guiado por fisiologia. Porém, o curto período de acompanhamento, o tamanho da amostra pequeno dos estudos incluídos e a não-discriminação das causas de morte podem justificar essas conclusões. Estudos com períodos mais longos de acompanhamento são necessários para tirar conclusões mais robustas e definitivas.

## Introdução

A angiografia coronária invasiva é considerada padrão ouro para o diagnóstico da doença arterial coronariana (DAC) envolvendo os vasos coronários epicárdicos.^1^ Porém, a avaliação visual por meio da angiografia coronária tradicional não é capaz de distinguir se uma estenose coronária é hemodinamicamente significativa, principalmente na DAC intermediária; então, há discordâncias constantes entre a gravidade angiográfica e a significância da fisiologia na DAC.^2,3^ As discrepâncias ocorrem porque, diferentemente da angiografia, a fisiologia incorpora os efeitos combinados e inter-relacionados do fluxo coronário e da resistência microvascular.^3^

Há crescentes evidências que apoiam os benefícios clínicos e a custo-efetividade da revascularização coronária percutânea guiada pela fisiologia, seja pelos índices baseados na hiperemia ou pelos índices baseados na diferença de pressão durante um período específico da diástole, em comparação à revascularização percutânea baseada na angiografia coronária.^4-7^ Porém, os resultados de longo prazo da avaliação da gravidade da estenose por meio da fisiologia, em comparação à angiografia como guia para a cirurgia de bypass, ainda são incertos.

Considerando esses resultados, cirurgiões cardíacos estão progressivamente guiando a revascularização da doença de múltiplos vasos (MVD) com base na fisiologia coronária. Porém, ainda não está claro se esta decisão traz melhores resultados clínicos de longo prazo. As recomendações sobre o uso do CABG em comparação à terapia médica ou à intervenção coronária percutânea (ICP) são inteiramente baseadas em estudos que utilizam critérios anatômicos e não-funcionais para guiar a revascularização.^8-12^

Por conta desses dados, muitos autores já avaliaram o potencial benefício clínico da cirurgia de bypass da artéria coronária guiada pela fisiologia, além dos detalhes anatômicos para decisões cirúrgicas.^13-20^ Neste artigo, estendemos o trabalho de Spadaccio et al.,^20^ ao agrupar todos os resultados de estudos randomizados e não-randomizados para avaliar o efeito nos resultados clínicos entre o CABG guiado por fisiologia em comparação ao CABG guiado pela angiografia.^20^

## Métodos

### Fontes de dados e buscas

Nós fizemos buscas sistemáticas nas bases Medline, Embase e Cochrane Library, pesquisando artigos publicados relevantes. A última data da busca foi em junho de 2020, e todos os estudos anteriores foram incluídos na busca. Revisões sistemáticas e qualitativas prévias, se disponíveis, foram consideradas para estudos adicionais. Os seguintes termos de busca foram utilizados: “Fisiologia coronária” ou “Fluxo Fracionado de Reserva” ou “FFR” ou “Instant Wave-Free Ratio” ou “iFR” ou “Enxerto de bypass de artéria coronária” ou “CABG”. Outros estudos foram buscados por pesquisa manual ou em fontes secundárias, incluindo referências de artigos primários. Não houve restrições de língua na busca.

### Seleção dos estudos

**Figura 1:**
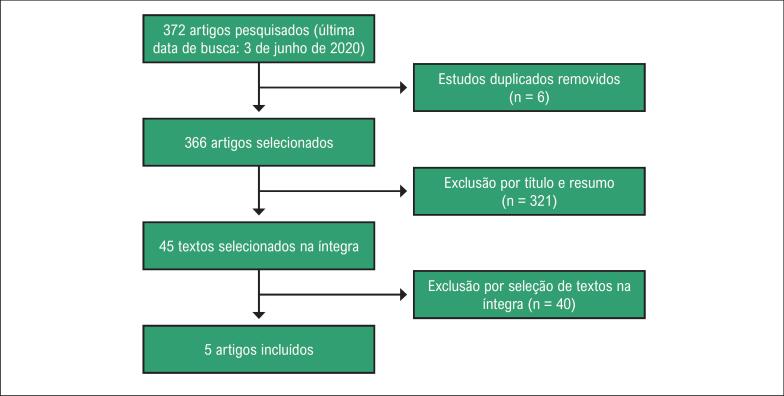
Fluxo dos estudos incluídos na metanálise.

As citações foram primeiramente selecionadas pelo título/resumo por dois revisores independentes (JM e LS), e os manuscritos completos foram avaliados se fossem potencialmente pertinentes. Discordâncias foram resolvidas após chegarem a um consenso. Os artigos identificados foram avaliados pelos mesmos revisores, de acordo com os seguintes critérios de inclusão: artigos com resultados clínicos comparando as duas estratégias para a revascularização no CABG (fisiologia *versus* angiografia). As disputas relacionadas aos critérios de inclusão foram resolvidas até chegarem a um consenso. Estudos comparando ambas as estratégias que não reportaram os resultados clínicos foram excluídos. Os estudos que não reportaram a estratégia usada em detalhe, embora tivessem a avaliação dos resultados clínicos, também foram excluídos (Figura 1).

### Desfechos

Os desfechos estudados foram: morte por todas as causas, infarto do miocárdio (IM), revascularização do vaso alvo (TVR) e eventos cardiovasculares adversos maiores (MACE) durante o período de acompanhamento.

MACE foi definido como um composto de morte, infarto do miocárdio ou qualquer revascularização de Moscona et al.,^16^ Fournier et al;^18^ e como um composto de morte, infarto do miocárdio, derrame ou qualquer revascularização dos estudos FARGO, FUTURE e GRAFFITI durante o período de acompanhamento.^13-19^

### Análise estatística

Para calcular as estimativas do efeito agrupado, utilizamos a variância inversa assumindo um modelo de efeito fixo, e o método de DerSimonian e Lard^21^ assumindo o modelo do efeito randômico.^21^ A homogeneidade entre os estudos foi avaliada com o teste Q de Cochrane e o índice I2 (os valores de 0,25, 0,50 e 0,75 indicaram níveis baixos, moderados e altos de heterogeneidade, respectivamente). O valor de p <0,05 foi considerado estatisticamente significativo. O viés de publicação foi avaliado utilizando o gráfico em funil. Realizamos uma análise de sensibilidade para mostrar o impacto de cada estudo nos resultados. O MetaXL 2.0 (EpiGear International Pty Ltd, Wilston, Queensland, Australia) foi usado para calcular o tamanho do efeito da diferença do risco agrupado (diferença entre o risco dos grupos com revascularização e com gerenciamento conservador).

## Resultados

### Identificação do Estudo

As buscas nas bases de dados inicialmente encontraram 372 citações. Dessas, seis estudos duplicados e 321 artigos foram excluídos após a revisão do título ou resumo. Depois de uma avaliação aprofundada de acordo com os critérios de seleção, excluímos mais 40 estudos. Um final total de cinco estudos foi incluído na análise. Esses cinco estudos incluíam 1.114 pacientes: 403 no grupo guiado pela fisiologia, e 711 no grupo guiado pela angiografia.

### Características dos estudos incluídos

Dos cinco estudos incluídos, três eram randomizados e dois eram não-randomizados, observacionais e retrospectivos (Tabelas 1 e 2)

**Tabela 1:** Características dos estudos incluídos

Autor	Ano	N total	N - estratégia	Acompanhamento	Desenho do estudo	Vias	Oclusão do enxerto - acompanhamento	Principais conclusões clínicas
Moscona et al.^16^	2018	109	Guiado por FFR/iFR-: 14 Guiado por angiografia: 95	18 meses	Retrospectivo	Via arterial: 92,9% (grupo FFR; 90,5% Grupo angiografia) EVS: 85,7% (grupo FFR;76,8%; grupo angiografia)	NR	Uma tendência de redução da MACE (7,1% *vs.* 11,6%, P=0,369) e angina (0,0% *vs.* 6,3%, P=0,429) no grupo FFR/iFR em comparação ao grupo da angiografia.
Thuesen et al.^14^	2018	97	Guiado por FFR: 49 Guiado por angiografia: 48	Seis meses	Estudo controlado randomizado	Via arterial: 37%	Falência de enxerto de todos os enxertos foi similar em ambos os grupos (16% vs. 12%; p = 0,97).	Taxas de morte, IM, e derrame foram similares nos grupos do estudo. A mortalidade por todas as causas após seis meses foi de 0% no grupo FFR, e 4,1% no grupo angiografia; um paciente morreu por conta de embolia pulmonar, e um morreu de mediastinite.
Rioufol et al.^17^	2018	109	Guiado por FFR: 55 Guiado por angiografia: 54	Um ano	Estudo randomizado controlado	NR	NR	FFR em pacientes com doença de múltiplos vasos não demonstrou melhora no desfecho do composto primário de mortalidade por todas as causas, IM, revascularização repetida ou derrame por um ano (14,6% vs 14,4%; HR 0,97; IC95% 0,69-1,36) Risco de morte foi significativamente maior no grupo FFR (3,7% vs 1,5%; *P* = 0,036)
Fournier et al.^15^	2019	627	Guiado por FFR: 198 Guiado por angiografia: 429	Seis anos	Retrospectivo	Via arterial: 64% EVS: 36%	A taxa de oclusão foi significativamente mais baixa no grupo FFR em comparação ao da angiografia	CABG guiado por FFR está associado à redução significative na taxa de morte geral ou infarto do miocárdio (HR 0,59 [IC95%, 0,38–0,93]; *P*=0,020)
Toth et al.^19^	2019	172	Guiado por FFR: 88 Guiado por angiografia: 84	Um ano	Estudo randomizado controlado	NR Taxa da Via arterial-para-EVS: 1:1	Não houve diferença na oclusão geral dos enxertos (80% vs 81%) p=0,885)	Não houve diferença no composto morte, infarto do miocárdio, vaso algo, revascularização e derrame (HR 1,275; IC95%: 0,391 a 4,160, *p*=0,674)

FFR: Fluxo fracionado de reserva; iFR: proporção instantânea de ondas livres; N: pacientes incluídos no estudo; IM: infarto do miocárdio; TVR: revascularização do vaso alvo; NR: não reportado; EVS: enxerto de veia safena; CABG: enxerto de bypass de artéria coronária. Valor de p < 0,05 foi considerado significativo em todos os estudos incluídos.

**Tabela 2:** Características dos estudos incluídos

Autor	Ano	N total	N - estratégia	Morte	IM	TVR	MACE
Moscona et al.^16^	2018	109	Guiado por FFR/iFR: 14 Guiado por angiografia: 95	FFR/iFR: 1 Angiografia: 5	FFR/iFR: 0 Angiografia: 2	FFR/iFR: 0 Angiografia: 4	FFR/iFR: 1 Angiografia: 11
Thuesen et al.^14^	2018	97	Guiado por FFR: 49 Guiado por angiografia: 48	FFR: 0 Angiografia: 2	FFR: 1 Angiografia: 0	FFR: 2 Angiografia: 0	FFR: 6 Angiografia: 6
Rioufol et al.^17^	2018	109	Guiado por FFR: 55 Guiado por angiografia: 54	FFR: 1 Angiografia: 0	NR	NR	HR 0.,45 (0,108-6,612)
Fournier et al.^15^	2019	627	Guiado por FFR: 198 Guiado por angiografia: 429	FFR: 21 Angiografia: 79	FFR: 11 Angiografia: 34	FFR: 17 Angiografia: 27	FFR: 42 Angiografia: 113
Toth et al.^19^	2019	172	Guiado por FFR: 88 Guiado por angiografia: 84	FFR: 3 Angiografia: 2	FFR: 0 Angiografia: 2	FFR: 2 Angiografia: 4	FFR: 5 Angiografia: 6

IM: infarto do miocárdio; TVR: revascularização do vaso alvo

### Síntese quantitativa dos desfechos

#### Morte por todas as causas.

**Figura 2:**
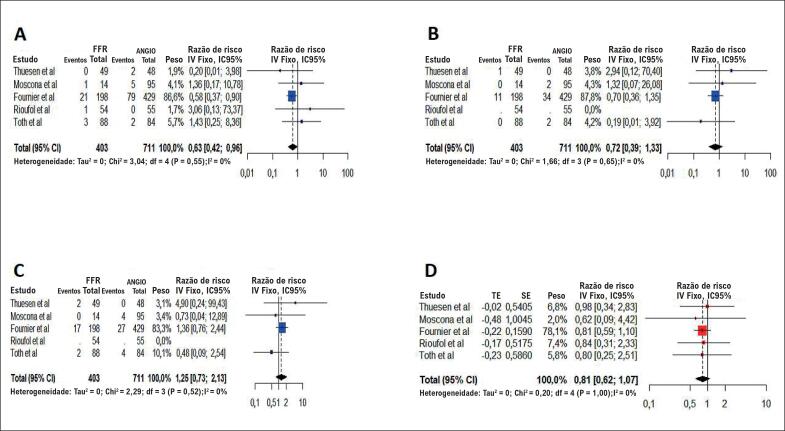
Gráfico em Floresta da razão de risco agrupada pelos desfechos: (A) morte por todas as causas; (B) IM; (C) TVR; (D) MACE. O tamanho dos marcadores de dados indica o peso do estudo. IC: intervalo de confiança; IM: infarto do miocárdio; TVR: revascularização do vaso alvo.

**Figura 3:**
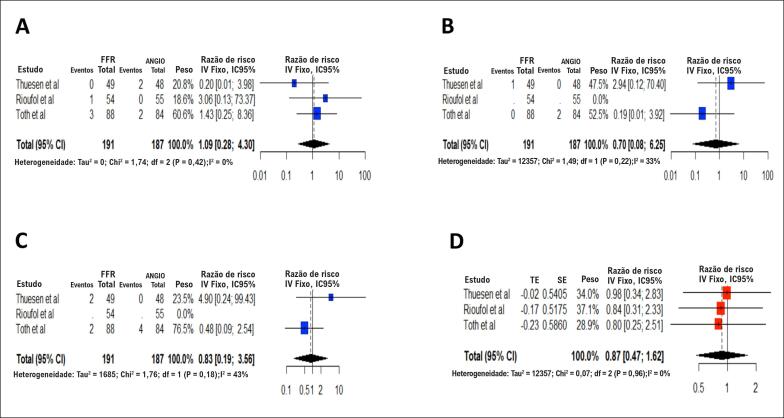
Gráfico em Floresta da razão de risco agrupada para os desfechos quando somente os estudos randomizados controlados foram incluídos: (A) morte por todas as causas; (B) IM; (C) TVR; (D) MACE. O tamanho dos marcadores de dados indica o peso do estudo. IC: intervalo de confiança; IM: infarto do miocárdio; TVR: revascularização do vaso alvo.

A morte por todas as causas foi reportada nos cinco estudos, o que foi considerado para a análise agrupada, para um total de 1.114 pacientes. A estratégia da fisiologia apresentou 0,63 vezes o risco de morte por todas as causas em comparação à estratégia da angiografia (RR = 0,63; IC95% = 0,42–0,96). A Figura 2 descreve a metanálise ponderada para a morte por todas as causas. A análise agrupada demonstrou heterogeneidade insignificante entre os estudos (I2 = 0%; p = 0,55). Em uma análise de sensibilidade, ao recalcular os resultados agrupados da análise primária ao excluir cada estudo por vez, no estudo de Fournier et al. esta diferença de risco desaparece. Este efeito também desapareceu quando limitamos a análise a estudos randomizados controlados (RR = 1,09; IC95% = 0,28–4,3). A Figura 3 descreve a metanálise ponderada para a morte por todas as causas quando só os estudos randomizados controlados foram incluídos.

#### Infarto do miocárdio.

Para analisar a ocorrência de IM, quatro estudos que incluíam um total de 1.093 pacientes foram agrupados. Não houve diferença significativa entre as duas estratégias (RR = 0,72; IC95% = 0,39–1,33), nem heterogeneidade significativa entre os estudos (I2 = 0%; p = 0,65). A exclusão de qualquer estudo único e dos não-randomizados não alterou o resultado combinado final.

#### Revascularização do vaso alvo.

Para avaliar a TVR, quatro estudos incluindo um total de 1.093 pacientes foram agrupados. Não houve diferença significativa entre as duas estratégias (RR = 1,25; IC95% = 0,73–2,13), nem heterogeneidade significativa entre os estudos (I2 = 0%; p = 0,55). A exclusão de qualquer estudo único e dos não-randomizados não alterou o resultado combinado final.

#### MACE.

Para avaliar os MACE, cinco estudos incluindo 1.114 pacientes foram agrupados. Não houve diferença significativa entre as duas estratégias (RR = 0,81; IC95% = 0,62–1,07), nem heterogeneidade significativa entre os estudos (I2 = 0%; p = 1). A exclusão de qualquer estudo único e dos não-randomizados não alterou o resultado combinado final.

### Viés do estudo

**Figura 4:**
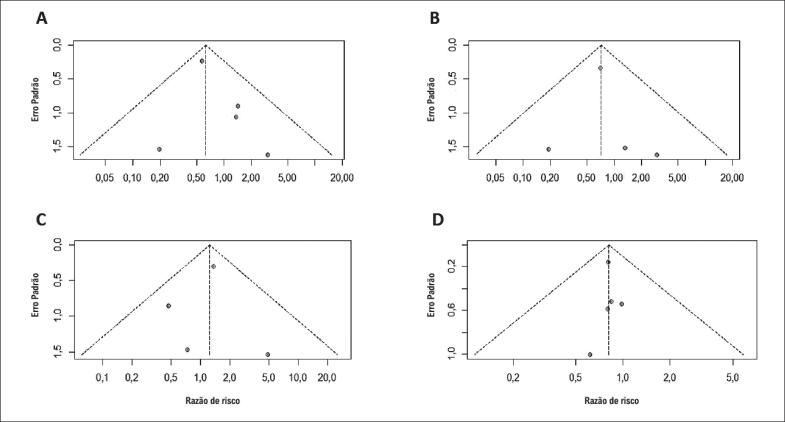
Viés de publicação para: (A) morte por todas as causas; (B) IM; (C) TVR; (D) MACE. Círculos representam estudos individuais da metanálise, e a linha vertical representa a estimativa agrupada da Razão de Risco para morte por todas as causas, IM, TVR e MACE. IM: infarto do miocárdio; TVR: revascularização do vaso alvo.

A inspeção visual do gráfico de funil para os desfechos não revelou nenhuma assimetria entre os estudos (Figura 4). Além disso, o teste de correlação de Begg não foi estatisticamente significante.

## Discussão

A sobrevivência tem uma correlação significativamente negativa com a carga obstrutiva da DAC angiográfica. O escore SYNTAX estratifica a complexidade angiográfica da doença arterial coronariana e estabelece o prognóstico de pacientes com MVD, sendo uma ferramenta importante para decidir sobre a melhor estratégia de revascularização.^22,23^

Há discordâncias entre a gravidade da significância da angiografia e da fisiologia da DAC, então, o escore SYNTAX, que é obtido ao considerar somente as lesões provocadas por isquemia, pode superar esta limitação. Em comparação com o clássico escore SYNTAX, o escore SYNTAX funcional tem melhor reprodutibilidade e valor prognóstico, reclassificando até 32% dos candidatos ao CABG e gerando implicações em termos de direcionamento terapêutico.^22-24^

Se os impactos favoráveis da fisiologia coronariana nos desfechos da ICP podem ou não ser traduzidos em prática cirúrgica tornou-se o tema de nossa investigação.

Nossa metanálise mostrou uma redução de 37% das mortes por todas as causas no grupo guiado pela fisiologia, com redução não estatisticamente significativa em IM e MACE; esses desfechos não estiveram associados ao aumento do TVR. Esses resultados devem ser interpretados considerando os limites intrínsecos a cada um dos estudos, incluindo viés de seleção, já que esta redução desaparece quando somente os estudos randomizados controlados são agrupados para análise.

Ao avaliar os desfechos clínicos na revascularização, há importantes considerações a serem feitas. A primeira é relacionada aos desfechos (peri) procedimento. O tipo de IM deve estar claramente estabelecido ao comparar as estratégias, já que hoje é universalmente aceito que o prognóstico do IM espontâneo não é similar ao IM periprocedimento ou o IM tipo 2.^25,26^

A história natural da doença é outro ponto importante a ser considerado como novo paradigma, com o foco na doença em si (aterosclerose) e não no sintoma (isquemia).^25,27^ A composição da placa, avaliada por algumas características da imagem, aparentemente é o principal determinante do prognóstico, até mais do que o nível da estenose coronária ou sua localização.^28-30^ Isso pode explicar o melhor prognóstico associado à completa revascularização no contexto da síndrome coronária aguda, na qual as placas em lesões não culpadas aparentemente possuem características instáveis, contrastando com os achados na doença coronariana estável.^31^

A terceira consideração importante é o tipo de revascularização. Os benefícios bem definidos do CABG em comparação à ICP guiada pela angiografia, como reportado nos estudos ASCERT, SYNTAX, FREEDOM e BEST, usam critérios anatômicos e não-funcionais para guiar a revascularização e vêm antes da tecnologia de stent farmacológico da nova geração.^8-12^

Uma diferença considerável entre o CABG e a ICP depende dos efeitos protetivos da progressão da doença aterosclerótica. Sabe-se que a maioria das estenoses relacionadas ao IM está localizada no terço proximal da árvore coronária. Também sabe-se que a maioria dos IMs advém de placas não-significantes. Enxertos cirúrgicos de bypass normalmente são implantados distalmente na circulação coronária, promovendo “um efeito de colateralização” na revascularização, e parece concebível que o benefício prognóstico do CABG possa ser explicado pela proteção contra eventos coronários, independentemente da gravidade da estenose do vaso enxertado. O conceito de revascularização baseado na fisiologia, e não no tipo de placa, elimina o efeito protetor do bypass cirúrgico.^28-30,32^

O conceito da completa revascularização surgiu de estudos prévios sobre o CABG, enquanto algumas publicações demonstravam que pacientes que estavam completamente revascularizados tinham um benefício em termos de mortalidade em relação àqueles que estavam revascularizados de forma incompleta, o que estabeleceu um padrão para o campo do CABG.^33-35^

A revascularização baseada na fisiologia também traz o conceito da revascularização completamente anatômica *versus* funcional. Se, por um lado, o uso da fisiologia coronária reduz o número de vasos enxertados, ou até de cirurgias, sem a circulação extracorpórea, por outro aumenta a taxa de revascularização incompleta anatomicamente definida.^19^

O uso da revascularização guiada pela fisiologia demonstrou reduzir a taxa de MACE nos pacientes com MVD, com ponto de corte de FFR de 0,78, mostrando associação significativa entre a medida de FFR pré-operatório do vaso alvo e a funcionalidade da anastomose após seis meses. Essas conclusões também são apoiadas por Botman et al.,^36^ para quem as lesões com FFR > 0,75 estão associadas ao aumento significativo do risco de oclusão do enxerto (p <0,0001).^18,19,36,37^

Considerando as diferenças descritas na suscetibilidade à competitividade de fluxo, parece provável que o tipo de conduta usada nos estudos FARGO e GRAFFITI (nos quais uma grande proporção dos enxertos eram enxerto de veia safena – EVS) *versus* o estudo IMPAG (no qual somente enxertos arteriais eram utilizados) possa explicar os resultados contraditórios.^14,19,38^

O estudo FAME 3 vai comparar, de forma multicêntrica e randomizada, a ICP guiada por FFR com stents farmacológicos contemporâneos com o CABG em pacientes com a doença de 3 vasos. Porém, não vai responder sobre a comparação entre o CABG guiado por fisiologia e o CABG guiado pela angiografia.^39^

Finalmente, o papel do acompanhamento neste contexto também deve ser considerado. Quanto mais grave a estenose coronária, maior o risco de IM, mas são as placas não-significativas as responsáveis pela maioria dos IMs. Também sabemos que a principal causa de morte nesses pacientes com DAC é relacionada ao coração, e que o IM é uma causa de morte cardíaca; então, as terapias para reduzir o IM ou a morte cardiovascular vão, consequentemente, reduzir a mortalidade.^32^

Podemos considerar o estudo STICH como exemplo, comparando o tratamento com terapia clínica somado ao CABG e somente a terapia clínica em pacientes com DAC e insuficiência cardíaca, com fração de ejeção reduzida. Após cinco anos de acompanhamento, a análise da intenção de tratamento não demonstrou diferenças significativas entre as duas estratégias com relação ao desfecho primário de morte por todas as causas. Porém, após o período de acompanhamento ter sido estendido para dez anos, uma redução significativa na mortalidade foi encontrada para o CABG somado à terapia clínica, em comparação à terapia clínica somente (HR, 0,84; IC95%: 0,73-0,97; p=0,02). Outro exemplo é o estudo FAME 2, no qual os dados publicados após cinco anos mostram uma forte tendência a baixas taxas de infarto do miocárdio no grupo ICP (HR 0,66; IC95% 0,43-1,00; p = 0,049), uma diferença que só foi significativa para o IM espontâneo (HR 0,62; IC95%; p = 0,04), e não para o IM periprocedimento. Recentemente, o estudo ISCHEMIA mostrou que, em um acompanhamento prévio, o desfecho composto primário (morte cardiovascular, IM ou hospitalização devido à angina instável ou insuficiência cardíaca) foi mais frequente no grupo com a estratégia invasiva do que no grupo da estratégia conservadora (5,3% vs. 3,4% após seis meses), devido aos IMs relacionados ao procedimento. Porém, em um acompanhamento posterior, depois de aproximadamente dois anos, as curvas dos eventos se cruzaram e, após cinco anos, a incidência do desfecho primário foi um pouco maior no grupo da estratégia conservadora (18,2% e 16,4%). Então, parece que para se ter um impacto nos desfechos duros, como morte por todas as causas, temos que estender a duração do período de observação.^25,40,41^

Em nosso estudo, a redução da morte por todas as causas em um contexto de redução não significativa do IM e MACE deve ser interpretada com cautela, já que esta situação pode estar relacionada a mortes causadas por motivos relacionados ao coração. Seria interessante não só avaliar se essas mortes têm relação com o coração, mas também estender o acompanhamento dos estudos para observar se as curvas dos desfechos duros divergem em períodos mais longos, possibilitando chegar a conclusões definitivas. É importante observar que somente o estudo de Fournier et al.,^18^ tem um período de acompanhamento mais longo do que cinco anos, o que pode explicar os resultados, já que a redução no desfecho composto morte por todas as causas e IM, apoiando a estratégia da fisiologia, só foi encontrada quando o período de acompanhamento foi estendido.^15^

### Limitações

As conclusões tiradas desta metanálise estão sujeitas às limitações e diferenças dos estudos originais incluídos na análise. A limitação desta metanálise é a presença de estudos com amostras menores e abrangentes, e resultados de sobrevivência de longo prazo. Outra limitação é representada por um viés de seleção intrínseco. Diversas decisões sobre revascularização na estratégia guiada pela fisiologia foram desviadas da indicação funcional nos estudos incluídos, e os autores justificaram que isso esteve relacionado a causas técnicas e, em alguns casos, à relutância para adiar a revascularização. Como mencionado, como a causa de morte não era conhecida, isso inclui mortes não relacionadas ao coração, ou seja, não ligadas à escolha da estratégia da revascularização. Outra limitação foi que com a inclusão de dois estudos retrospectivos e observacionais, alguns pacientes incluídos neste registro podem ter sido tratados com ICP guiada pela fisiologia, e não pelo CABG.

## Conclusão

Esta metanálise demonstra uma redução na morte por todas as causas quando a estratégia do CABG guiado pela fisiologia foi utilizada. Porém, o curto período de acompanhamento, a amostra pequena dos estudos incluídos e a não-discriminação de causas de morte podem justificar essas conclusões. Estudos com períodos mais longos de observação são necessários para se chegar a conclusões mais robustas e definitivas.
