# Supplementary material for: Enxerto de Bypass de Artéria Coronária Guiado por Angiografia ou Fisiologia: Uma Metanálise
Source: Arq Bras Cardiol. 2021 Nov 22;117(6):1115–23. [Article in Portuguese] doi: 10.36660/abc.20200763 (PMC8757150; doi:10.36660/abc.20200763)
Supplement: Translation

# Translation: Physiology or Angiography-Guided Coronary Artery Bypass Grafting: A Meta-Analysis

**Authors:** José Martins, Vera Afreixo, Luís Santos, Luís Fernandes, Ana Briosa

**Affiliations:** 1 Aveiro Portugal Baixo Vouga Hospital Centre, Aveiro - Portugal; 2 Aveiro Portugal CIDMA/IBIMED/Department of Mathematics, University of Aveiro, Aveiro - Portugal; 3 York United Kingdom Centre for Health Economics, University of York, York - United Kingdom

**Keywords:** Coronary Artery Disease, Angiography, Metanalysis, Coronary Artery/physiology, Coronary Angiography, Coronary Artery Bypass

## Abstract

**Background:**

While invasive coronary angiography is considered the gold standard for the diagnosis of coronary artery disease (CAD) involving the epicardial coronary vessels, coronary physiology-guided revascularization represents a contemporary gold-standard practice for the invasive management of patients with intermediate CAD. Nevertheless, the long-term results of assessing the severity of stenosis through physiology compared to the angiogram as the guide to bypass surgery – coronary artery bypass grafting (CABG) are still uncertain. This meta-analysis aims to assess the clinical outcomes of a physiology guided CABG compared to the angiography-guided CABG.

**Objectives:**

We sought to determine if outcomes differ between a physiology guided CABG compared to an angiography-guided CABG.

**Methods:**

We searched Medline, EMBASE, and the Cochrane Library. The last date for this search was June 2020, and all of the previous studies were included. We conducted a pooled risk-ratio meta-analysis for four main outcomes: all-cause death, myocardial infarction (MI), target vessel revascularization (TVR) and major adverse cardiovascular events (MACE). P-value <0.05 was considered as statistically significant. Heterogeneity was assessed with Cochran's Q test and quantified by the I^2^ index.

**Results:**

We identified five studies that included a total of 1,114 patients. A pooled meta-analysis showed no significant difference between a physiology guided strategy and an angiography-guided strategy in MI (risk ratio [RR] = 0.72; 95%CI, 0.39–1.33; I2 = 0%; p = 0.65), TVR (RR = 1.25; 95%CI = 0.73–2.13; I2 = 0%; p = 0.52), or MACE (RR = 0.81; 95%CI = 0.62–1.07; I2 = 0%; p = 1). The physiology guided strategy has 0.63 times the risk of all-cause death compared to the angiography-guided strategy (RR = 0.63; 95%CI = 0.42–0.96; I2 = 0%; p = 0.55).

**Conclusion:**

This meta-analysis demonstrated a reduction in all-cause death when a physiology guided CABG strategy was used. Nevertheless, the short follow-up period, small sample size of the included studies and the non-discrimination of the causes of death can largely justify these conclusions. Studies with an extended follow-up period of observation are required to draw more robust and definitive conclusions.

## Introduction

Invasive coronary angiography is considered as the *gold standard* for the diagnosis of coronary artery disease (CAD) involving the epicardial coronary vessels.^1^ Nevertheless, visual assessment by traditional coronary angiography is unable to distinguish if a coronary stenosis is hemodynamically significant, particularly in intermediate CAD, so there are often discordances between the angiographic severity and the physiological significance of CAD.^2,3^ Discrepancies occur because, unlike angiography, physiology incorporates the combined and inter-related effects of coronary flow and microvascular resistance.^3^

There is growing evidence supporting the clinical benefit and cost-effectiveness of coronary physiology guided percutaneous revascularization, either by indices based on hyperemia or indices based on pressure difference during a specific period in diastole, compared to percutaneous revascularization based on coronary angiography.^4-7^ However, the long-term results of assessing the severity of stenosis through physiology compared to the angiogram as the guide to bypass surgery are still uncertain.

Cardiac surgeons are, in the light of the results, progressively guiding multivessel disease (MVD) revascularization based on coronary physiology. However, whether this decision entails better long-term clinical outcomes or not is still unclear. Recommendations on the use of CABG compared with medical therapy or percutaneous coronary intervention (PCI) are entirely based on studies that used anatomical and non-functional criteria to guide revascularization.^8-12^

Given this data, several authors have already evaluated the potential clinical benefit of physiology guided coronary bypass surgery in addition to anatomic detail for surgical decisions.^13-20^ In this article, we extend the work of Spadaccio et al.^20^ by pooling all results from randomized and non-randomized studies to assess the effect on clinical outcomes between a physiology guided CABG compared to angiography-guided CABG.^20^

## Methods

### Data sources and searches

We systematically searched Medline, Embase, and the Cochrane Library for relevant published articles. The last date for this search was June 2020, and all of the previous studies were included in the search. Previous qualitative and systematic reviews, if available, were checked for additional studies. The following query term was used: “Coronary physiology” or “Fractional Flow Reserve” or “FFR” or “Instant Wave-Free Ratio” or “iFR” or “Coronary Artery Bypass Graft” or “CABG”. Further studies were sought by means of a manual search of secondary sources, including references from primary articles. No language restrictions were enforced.

### Study selection

Citations were first screened at the title/abstract level by two independent reviewers (JM and LS), and complete manuscripts were retrieved if potentially pertinent. Disagreements were resolved after consensus. The identified articles were independently appraised by the same reviewers according to the following inclusion criteria: articles with clinical outcomes comparing the two strategies for CABG revascularization (physiology vs angiography). Disputes regarding the inclusion criteria were resolved by consensus. Studies comparing the two strategies that did not report clinical outcomes were excluded. Studies that, despite the evaluation of clinical outcomes, did not report the strategy used in detail, were also excluded (Figure 1).

**Figure 1:** Flow chart of studies included in the meta-analysis.

### Endpoints

The studied endpoints were: all-cause death, myocardial infarction (MI), target vessel revascularization (TVR) and major adverse cardiovascular events (MACE) during the follow-up period.

MACE was defined as a composite of death, myocardial infarction, or any revascularization by Moscona et al.,^16^ Fournier et al.^18^ and as composite of death, myocardial infarction, stroke, or any revascularization in the FARGO, FUTURE and GRAFFITI studies during the follow-up period.^13-19^

### Statistical analysis

To calculate the pooled effect estimates, we used the inverse variance assuming a fixed-effect model and the DerSimonian and Laird method assuming a random-effect model.^21^ Homogeneity among the studies was evaluated using the Cochran's Q test and the I2 statistic (the values of 0.25, 0.50, and 0.75 indicated low, moderate, and high levels of heterogeneity, respectively). *P-*value <0.05 was considered statistically significant. Publication bias was evaluated using the funnel plot. We performed a sensitivity analysis to show the impact of each study on the results. MetaXL 2.0 (EpiGear International Pty Ltd, Wilston, Queensland, Australia) was used to calculate the pooled risk difference effect size (difference between the risk between revascularization and conservative management groups).

## Results

### Study identification

Database searches initially retrieved 372 citations. Of these, 6 duplicate studies and 321 articles were excluded after the review of the title or abstract. After a thorough assessment according to the selection criteria, we further excluded 40 studies. A final total of five studies were included in the analysis. These five studies included 1,114 patients: 403 in the physiology guided group and 711 the angiography-guided group.

### Characteristics of the included studies

Of the five included studies, three were randomized and two were non-randomized, observational, and retrospective in design (Tables 1 and 2).

**Table 1:** Characteristics of the included studies

Author	Year	N total	N Strategy	FU	Study design	Conduits	Graft patency FU	Major clinical conclusions
Moscona et al.^16^	2018	109	FFR/iFR-guided: 14 Angiography-guided: 95	18 months	Retrospective	Arterial conduits: 92.9% (FFR- group; 90.5% Angiography-group) SVG: 85.7% (FFR- group;76.8% Angiography-group)	NR	A trend toward reduction in MACE (7.1% *vs.* 11.6%, P=0.369) and angina (0.0% *vs.* 6.3%, P=0.429) in the FFR/iFR group compared to the angiography group
Thuesen et al.^14^	2018	97	FFR-guided: 49 Angiography-guided: 48	Six months	RCT	Arterial conduits: 37%	Graft failures of all grafts were similar in both groups (16% vs. 12%; p = 0.97).	Rates of death, MI, and stroke were similar in the study groups. All-cause mortality at six months was 0% in the FFR-guided group and 4.1% in the angiography-guided group; one patient died because of pulmonary embolism, and one died because of mediastinitis
Rioufol et al.^17^	2018	109	FFR-guided: 55 Angiography-guided: 54	One year	RCT	NR	NR	FFR in all-comer multivessel-disease patients did not demonstrate any improvement in the primary composite endpoint composite of all-cause mortality, MI, repeat revascularization, or stroke through one year (14.6% vs 14.4%; HR 0.97; 95%CI 0.69-1.36) Risk of death was significantly higher in the FFR-guided arm (3.7% vs 1.5%; *P* = 0.036)
Fournier et al.^15^	2019	627	FFR-guided: 198 Angiography-guided: 429	Six years	Retrospective	Arterial conduits: 64% SVG: 36%	The occlusion rate was significantly lower with FFR-guided as compared with angiography-guided grafts	FFR-guided CABG is associated with a significant reduction in the rate of overall death or myocardial infarction (HR 0.59 [95% CI, 0.38–0.93]; *P*=0.020)
Toth et al.^19^	2019	172	FFR-guided: 88 Angiography-guided: 84	One year	RCT	NR Arterial conduits-to-SVG ratio: 1:1	No difference in overall graft patency (80% vs 81%) p=0.885)	No difference in the composite of death, myocardial infarction, target vessel revascularization and stroke (HR 1.275; 95% CI: 0.391 to 4.160, *p*=0.674)

FU: follow-up; FFR: Fractional flow reserve; iFR: instant wave-free ratio; N: patients included in the study; MI: myocardial infarction; TVR: target vessel revascularization; NR: not reported; RCT: randomized controlled trial; SVG: saphenous vein graft; CABG: coronary artery bypass grafting. A value of p < 0.05 was considered significant in all included studies.

**Table 2:** Characteristics of the included studies

Author	Year	N total	N Strategy	Death	MI	TVR	MACE
Moscona et al.^16^	2018	109	FFR/iFR-guided: 14 Angiography-guided: 95	FFR/iFR: 1 Angiography: 5	FFR/iFR: 0 Angiography: 2	FFR/iFR: 0 Angiography: 4	FFR/iFR: 1 Angiography: 11
Thuesen et al.^14^	2018	97	FFR-guided: 49 Angiography-guided: 48	FFR: 0 Angiography: 2	FFR: 1 Angiography: 0	FFR: 2 Angiography: 0	FFR: 6 Angiography: 6
Rioufol et al.^17^	2018	109	FFR-guided: 55 Angiography-guided: 54	FFR: 1 Angiography: 0	NR	NR	HR 0.845 (0.108-6.612)
Fournier et al.^15^	2019	627	FFR-guided: 198 Angiography-guided: 429	FFR: 21 Angiography: 79	FFR: 11 Angiography: 34	FFR: 17 Angiography: 27	FFR: 42 Angiography: 113
Toth et al.^19^	2019	172	FFR-guided: 88 Angiography-guided: 84	FFR: 3 Angiography: 2	FFR: 0 Angiography: 2	FFR: 2 Angiography: 4	FFR: 5 Angiography: 6

MI: myocardial infarction; TVR: target vessel revascularization.

### Quantitative synthesis of outcomes

#### All-cause death.

All-cause death was reported in five studies, which we considered for pooled analysis, for a total of 1,114 patients. The physiology guided strategy has 0.63 times the risk of all-cause death compared to the angiography-guided strategy (RR = 0.63; 95%CI = 0.42–0.96). Figure 2 describes the weighted meta-analysis for all-cause death. The pooled analysis showed negligible heterogeneity among the studies (I2 = 0%; p = 0.55). Under a sensitivity analysis, by recalculating the pooled results of the primary analysis by excluding each single study in turn, in the study by Fournier et al. this risk difference disappears. This effect also disappeared when we limited the analysis to Randomized Controlled Trials (RCTs) (RR = 1.09; 95%CI = 0.28–4.3). Figure 3 describes the weighted meta-analysis for all-cause death when only RCTs were included.

**Figure 2:** Forest plot of the pooled risk ratio for the outcomes: (A) all cause death; (B) MI; (C) TVR; (D) MACE. The sizes of data markers indicate the weight of the study. CI: confidence interval; MI: myocardial infarction; TVR: target vessel revascularization.

**Figure 3:** Forest plot of the pooled risk ratio for the outcomes when only RCTs were included: (A) all-cause death; (B) MI; (C) TVR; (D) MACE. The sizes of data markers indicate the weight of the study. CI: confidence interval; MI: myocardial infarction; TVR: target vessel revascularization.

#### Myocardial infarction.

To analyze the occurrence of MI, four studies that included a total of 1,093 patients were pooled. No significant difference was noted between the two strategies (RR = 0.72; 95%CI = 0.39–1.33) and there was no significant heterogeneity among the studies (I2 = 0%; p = 0.65). The exclusion of any single study and the non-RCTs did not alter the overall combined result.

#### Target vessel revascularization.

To analyze TVR, four studies that included a total of 1,093 patients were pooled. No significant difference was noted between the two strategies (RR = 1.25; 95%CI = 0.73–2.13) and there was no significant heterogeneity among the studies (I2 = 0%; p = 0.55). The exclusion of any single study and the non-RCTs did not alter the overall combined result.

#### MACE.

To analyze MACE, five studies that included a total of 1,114 patients were pooled. No significant difference was noted between the two strategies (RR = 0.81; 95%CI = 0.62–1.07) and there was no significant heterogeneity among the studies (I2 = 0%; p = 1). The exclusion of any single study and the non-RCTs did not alter the overall combined result.

### Study Bias

The visual inspection of the funnel plot for the outcomes did not reveal any asymmetry among the studies (Figure 4). Further, the Begg's rank correlation test was not statistically significant.

**Figure 4:** Publication bias for: (A) all-cause death; (B) MI; (C) TVR; (D) MACE. Circles represent individual studies of the meta-analysis and the vertical line represents the pooled estimate of the Risk Ratio for all cause death, MI, TVR and MACE. MI: myocardial infarction; TVR: target vessel revascularization.

## Discussion

Survival has a significant negative correlation with the burden of angiographic obstructive CAD. The SYNTAX score stratifies the angiographic complexity of coronary artery disease and establishes the prognosis of patients with MVD, being an important tool to decide on the best revascularization strategy.^22,23^

There are often discordances between the severity of angiography and the physiological significance of CAD, so the functional SYNTAX score, which is obtained by counting only ischemia-provoking lesions, can overcome this limitation. Compared with the classic SYNTAX score, the functional SYNTAX score has better reproducibility and prognostic value, reclassifying up to 32% of CABG candidates, with implications in terms of the therapeutic guidance that this entails.^22-24^

Whether or not the favorable impact of coronary physiology on PCI outcomes could be translated to surgical practice became the subject of our investigation.

Our meta-analysis showed a 37% reduction in all-cause death in the group guided by physiology, with a non-statistically significant reduction in MI and MACE; these outcomes were not associated with an increase in TVR. These results must be interpreted within the limitations intrinsic to each of the studies, including selection bias, since this reduction disappears when only RCTs have been pooled for analysis.

When clinical outcomes in revascularization are evaluated, important considerations must be addressed. The first consideration to take into account is related to the (peri) procedural outcomes. The type of MI must be clearly established when comparing strategies, as it is now universally accepted that the prognosis of spontaneous MI is not similar to peri-procedural MI or type 2 MI.^25,26^

The natural history of the disease is another important point to consider as a new paradigm focusing on the disease itself (atherosclerosis), rand not on the symptom (ischemia). ^25,27^ The composition of the plaque, evaluated by certain imaging features, seems to be the main determinant of prognosis, more than the level of coronary stenosis or its location.^28-30^ This may explain the better prognosis associated with complete revascularization in the context of acute coronary syndrome, in which plaques in non-culprit lesions appear to have unstable characteristics, in contrast to what is found in stable coronary disease.^31^

The third important consideration is the type of revascularization. The well-defined benefits of CABG compared with angiography-guided PCI as reported in the ASCERT, SYNTAX, FREEDOM and BEST trials used anatomical and non-functional criteria to guide revascularization and are prior to the newer generation drug-eluting stent technology.^8-12^

An important difference between CABG and PCI depends on the protective effects of atherosclerotic disease progression. It is known that most stenoses related to MI are located in the proximal third of the coronary tree. We also know that most MIs arise from non-significant plaques. Surgical bypass grafts are usually implanted distally in the coronary circulation, providing “a collateralization effect” on revascularization, and it seems conceivable that the prognostic benefit of CABG can be explained by protection against coronary events, regardless of the severity of the stenosis of the grafted vessel. The concept of revascularization based on physiology, and not on the type of plaque, eliminates the protective effect of the surgical bypass.^28-30,32^

The concept of complete revascularization arose from the early studies on CABG, whereas some publications demonstrated that patients who were completely revascularized experienced a mortality benefit over those who were incompletely revascularized, thus setting the standard for the field of CABG.^33-35^

The revascularization based on physiology also raises the concept of complete anatomical vs. functional revascularization. If, on the one hand, the use of coronary physiology reduces the number of grafted vessels, or even surgery, without extracorporeal circulation, on the other hand, it increases the rate of anatomically defined incomplete revascularization.^19^

The use of physiology guided revascularization has been shown to reduce the MACE rate in patients with MVD, with the FFR 0.78 cut-off point showing a significant association between the preoperative FFR measurement of the target vessel and the anastomotic functionality at six months. These conclusions are also supported by Botman et al.,^36^ for whom lesions with FFR> 0.75 are associated with a significant increase in the risk of graft occlusion (p <0.0001).^18,19,36,37^

In light of the differences described in susceptibility to competitive flow, it seems likely that the type of conduit used in the FARGO and GRAFFITI trials (in which a large proportion of grafts were SVGs) versus IMPAG (in which only arterial grafts were used) may explain the contradictory results.^14,19,38^

The FAME 3 study will compare in a multicenter, randomized fashion FFR-guided PCI with contemporary drug-eluting stents to CABG in patients with 3-vessel coronary artery disease. It will not answer, however, the question of physiology guided CABG compared to angiography-guided CABG.^39^

Finally, the role of follow-up in this context would also be interesting to consider. The more severe the coronary stenosis, the higher the risk of MI, but it is the non-significant plaques that are responsible for most MIs. We also know that the main cause of death in these patients with CAD is cardiac-related, and that MI is a cause of cardiac death, so therapies that reduce the MI or cardiovascular death will subsequently decrease mortality.^32^

We should take the STICH trial as an example, comparing treatment with medical therapy plus CABG and medical therapy alone in patients with CAD and heart failure, with reduced ejection fraction. At five years of follow-up, the intention-to-treat analysis demonstrated no significant difference between the two strategies with respect to the primary outcome of all-cause death. However, after the follow-up period was extended to ten years, a significant reduction in mortality was found for CABG plus medical therapy compared to medical therapy alone (HR, 0.84; 95%CI: 0.73-0.97; p=0.02). Another example is the FAME 2 trial, in which the data published at five years show a strong tendency of lower rates of myocardial infarction in the PCI group (HR 0.66; 95%CI 0.43-1.00; *p* = 0.049), a difference that was only significant for spontaneous MI (HR 0.62; 95%CI; *p* = 0.04), not periprocedural MI. Recently, the ISCHEMIA trial showed that, in early follow-up, the primary composite outcome (cardiovascular death, MI, or hospitalization for unstable angina or heart failure) was more frequent in the invasive strategy group than in the conservative strategy group (5.3% vs. 3.4% at six months), due to procedure-related MI's. However, in a posterior follow-up, after about two years, the event curves crossed, and at five years, the incidence of the primary outcome was slightly higher in the conservative strategy group (18.2% and 16.4%). So, it seems that in order to have an impact on hard outcomes, like all-cause death, we have to extend the duration of the observation period.^25,40,41^

In our study, the reduction of all-cause death in a context of non-significant reduction of MI and MACE should be interpreted with caution, since this situation could be owed to deaths from non-cardiac causes. It would be interesting not only to assess if these deaths were cardiac-related, but also to extend the follow-up of the studies to observe if the curves of the hard outcomes diverge on longer follow-up periods, allowing to reach definitive conclusions. It should be noted that only the study by Fournier et al.^18^ has a follow-up period longer than five years, which can explain the results, since the reduction in the composite endpoint all-cause death or MI supporting a physiology guided strategy was only found when the follow-up was extended.^15^

### Limitations

The conclusions drawn from this meta-analysis are subjected to the limitations and differences of the original studies included in the analysis. A limitation of this meta-analysis is the presence of trials with small samples and wide-ranging, long-term survival results. Another limitation is represented by an intrinsic selection bias. Several revascularization decisions in the physiology guided strategy were deviated from the functional indication in the included trials, justified by some authors to be related to technical causes and, in some cases, the reluctance to defer revascularization. As aforementioned, as the cause of death was not known, this includes non-cardiac deaths unrelated to the choice of revascularization strategy. Another limitation was that with the inclusion of two retrospective and observational studies, some patients included in this registry might have been treated with physiology guided PCI instead of CABG.

## Conclusion

This meta-analysis demonstrates a reduction in all-cause death when a physiology guided CABG strategy was used. Nevertheless, the short follow-up period, small sample size of the included studies and the non-discrimination of the causes of death can largely justify these conclusions. Studies with an extended follow-up observation period are required to reach more robust and definitive conclusions.
